# Inorganic Polyphosphate, Exopolyphosphatase, and *Pho84*-Like Transporters May Be Involved in Copper Resistance in *Metallosphaera sedula* DSM 5348^T^

**DOI:** 10.1155/2018/5251061

**Published:** 2018-03-05

**Authors:** Matías Rivero, Constanza Torres-Paris, Rodrigo Muñoz, Ricardo Cabrera, Claudio A. Navarro, Carlos A. Jerez

**Affiliations:** ^1^Laboratory of Molecular Microbiology and Biotechnology, Faculty of Sciences, University of Chile, Santiago, Chile; ^2^Laboratory of Biochemistry and Molecular Biology, Faculty of Sciences, University of Chile, Santiago, Chile

## Abstract

Polyphosphates (PolyP) are linear polymers of orthophosphate residues that have been proposed to participate in metal resistance in bacteria and archaea. In addition of having a CopA/CopB copper efflux system, the thermoacidophilic archaeon *Metallosphaera sedula* contains electron-dense PolyP-like granules and a putative exopolyphosphatase (PPX*_Msed_*, *Msed_0891*) and four presumed *pho84*-like phosphate transporters (*Msed_0846*, *Msed_0866*, *Msed_1094*, and *Msed_1512*) encoded in its genome. In the present report, the existence of a possible PolyP-based copper-resistance mechanism in *M. sedula* DSM 5348^T^ was evaluated. *M. sedula* DSM 5348^T^ accumulated high levels of phosphorous in the form of granules, and its growth was affected in the presence of 16 mM copper. PolyP levels were highly reduced after the archaeon was subjected to an 8 mM CuSO_4_ shift. PPX*_Msed_* was purified, and the enzyme was found to hydrolyze PolyP *in vitro*. Essential residues for catalysis of PPX*_Msed_* were E111 and E113 as shown by a site-directed mutagenesis of the implied residues. Furthermore, *M. sedula ppx*, *pho84*-like, and *copTMA* genes were upregulated upon copper exposure, as determined by qRT-PCR analysis. The results obtained support the existence of a PolyP-dependent copper-resistance system that may be of great importance in the adaptation of this thermoacidophilic archaeon to its harsh environment.

## 1. Introduction

Acid mine drainage (AMD) and acid rock drainage (ARD) are the major environmental problems caused by biomining. Diverse biotic and abiotic strategies have been developed to treat AMD [1]. Bioremediation of metal-polluted soils consists of two steps: (i) the solubilization of metals from the matrix into the liquid phase and (ii) the extraction and concentration of metals [2]. Consortiums of thermophilic bacteria have been proposed as possible candidates for bioremediation of metal-contaminated sites due to their capacity to adsorb metals [3]. Thermoacidophilic archaea, such as *Sulfolobus metallicus* and *Acidianus brierleyi*, are important microorganisms used in bioleaching and can live in the hostile environmental conditions present in AMD. These archaea are iron and sulfur oxidizers, living in acidic and high-temperature environments (>60°C), and are generally very resistant to high metal concentrations [4]. Although these unique characteristics make thermoacidophiles possible efficient candidates for bioremediation of AMD, so far no strategies have been proposed using these microorganisms. A better understanding of how thermoacidophilic archaea can survive in AMD-conditions is still needed [5].

*Metallosphaera sedula* is a thermoacidophilic archaeon, first isolated from a continental solfataric field in Italy [6]. This microorganism is able to grow heterotrophically, autotrophically, and mixotrophically [7] and at high concentrations of metals such as copper [6]. Genomic analyses of strain DSM 5348^T^ [8] revealed some genes that could be involved in copper resistance in this microorganism. A P-type ATPase CopA *(Msed_0490)*, a metallochaperone CopM *(Msed_0491)*, and a transcriptional regulator CopT *(Msed_0492)* constitute a functional copper efflux system [9]. Cross-species complementation of a copper-sensitive *Sulfolobus solfataricus copR* mutant with these genes from *M. sedula* DSM 5348^T^ increased its copper resistance by 2-fold. Interestingly, copper resistance of a *M. sedula* strain mutant in *copA* was almost 2 times reduced compared to the wild-type strain. However, the resistance of *M. sedula copA* mutant was 4 times higher than that of *S. solfataricus copR* strain complemented with *copTMA* genes from *M. sedula*. That residual resistance present in the *M. sedula copA* mutant indicates that this archaeon may possess additional determinants to cope with copper stress [9].

Another possible metal resistance mechanism that has been proposed for the acidophilic *S. metallicus* and *Acidithiobacillus ferrooxidans* involves inorganic polyphosphate (PolyP) [10, 11]. These inorganic molecules are lineal polymers of orthophosphate residues linked by anhydride bonds [12]. In some acidophilic microorganisms, PolyP accumulates in the form of granules in the cytoplasm and it is rapidly hydrolyzed to inorganic phosphate (P_i_) after copper exposure [13]. The enzyme that synthesizes these polymers (“polyP polymerase”) has not yet been described for Crenarchaeota [4]. The exopolyphosphatase (PPX) catalyzes the hydrolysis of the terminal P_i_ residue of PolyP in *E. coli* [14], and a functional archaeal PPX has been characterized in *S. solfataricus* [15]. It has been suggested that metals could form metal-phosphate complexes that might be transported out of the cell by H^+^-phosphate symporters [16, 17]. In *E. coli*, P_i_ was expelled from the cell by a PitA H^+^-phosphate symporter following a copper shock, immediately after PolyP was degraded [18]. However, the PitA-based mechanism seems not to be ubiquitous, since other species, such as *A. ferrooxidans* and *S. metallicus*, lack orthologous genes to *pitA* and P_i_ is also supposed to be exported after a copper shift in those species. It has been proposed that a putative *pho84*-like gene, homolog to the yeast *Pho84* H^+^-phosphate symporter, could carry out that role [10].

*M. sedula* DSM 5348^T^ also accumulates electron-dense granules most likely composed of PolyP [13], and its gene *Msed_0891* encodes for a putative PPX*_Msed_* [8]. Although only a fragment of a PitA homolog is encoded in its genome, it contains four genes coding for putative *Pho84*-like phosphate transporters: *Msed_0846*, *Msed_0866*, *Msed_1094*, and *Msed_1512* [8]. It is possible that these proteins carry out the role of the lacking PitA in a putative PolyP-based copper-resistance mechanism in this strain. Recently, an extremely high copper-resistant (>200 mM) *M. sedula* strain CuR1 was isolated [9]. The sequencing of its genome revealed that a frameshift mutation had restored a complete *pitA* gene, encoded partially by the pseudogene *Msed_1517* of strain DSM 5348^T^ [19]. Those findings suggested that *M. sedula* could also have a copper-resistance mechanism involving PolyP, the putative PPX gene, and H^+^-phosphate symporters. In the present report, the PPX*_Msed_* enzyme from *M. sedula* DSM 5348^T^ was purified and partially characterized in its capacity to hydrolyze PolyP. The effects of copper on growth and PolyP levels, and the increased transcriptional levels of *ppx*, *copTMA*, and the four *pho84*-like genes in the presence of copper, further support the existence of a PolyP-dependent copper-resistance mechanism in the archaeon *M. sedula* DSM 5348^T^.

## 2. Materials and Methods

### 2.1. Archaeal and Bacterial Strains and Growth Conditions

*M. sedula* strain DSM 5348^T^ was grown at 65°C in DSMZ medium 88 (containing in g/L: 1.3 (NH_4_)_2_SO_4_, 0.28 KH_2_PO_4_, 0.25 MgSO_4_·7H_2_O, 0.07 CaCl_2_·2H_2_O, and 0.02 FeCl_3_·6H_2_O and in mg/L: 1.8 MnCl_2_·4H_2_O, 4.5 Na_2_B_4_O_7_·10H_2_O, 0.22 ZnSO_4_·7H_2_O, 0.05 CuCl_2_·2H_2_O, 0.03 Na_2_MoO_4_·2H_2_O, 0.03 VOSO_4_·2H_2_O, 0.01 CoSO_4_ and 0.1% yeast extract, and pH 2.0).

Four different growth conditions were analyzed: (i) “control”: microorganisms were grown in DSMZ medium 88 with no other additions until late exponential phase. (ii) “Nonadapted”: microorganisms never exposed to copper before were grown in DSMZ medium 88 and were added to 8 mM CuSO_4_ upon inoculation. (iii) “Adapted”: microorganisms were grown in DSMZ medium 88 supplemented with increasing copper concentrations (2, 4, 8, 12, and up to 16 mM). Thereafter, adapted cells were grown at 16 mM copper upon inoculation. (iv) “Shift”: microorganisms grown in DSMZ medium 88 up to the late exponential phase were abruptly exposed to 8 mM CuSO_4_ in the same medium.

Commercial *E. coli* TOP 10 (Invitrogen) and BL21 (DE3) strains were used for cloning and protein expression, respectively. These transformed bacteria were grown at 37°C in Luria-Bertani (LB) medium supplemented with 50 *μ*g/mL kanamycin. For solid plate cultures, 1.5% agar was added to the medium.

### 2.2. qRT-PCR

*M. sedula* DSM 5348^T^ cells were collected by centrifugation, washed twice with 10 mL of M88 medium, and resuspended in 60 *μ*L of TEN buffer (20 mM Tris-HCl pH 8.0, 1 mM EDTA, and 100 mM NaCl). Sixty *μ*L of TENST buffer (20 mM Tris-HCl pH 8.0, 1 mM EDTA, 100 mM NaCl, 1.6% sodium n-lauroylsarcosine, and 0.12% Triton X-100) was added to the suspension before RNA extraction. Total RNA from *M. sedula* was extracted with TRIzol Reagent (Ambion) and cDNA was synthesized as described previously [20, 21]. Transcriptional gene expression levels were quantified by qRT-PCR as described before [22]. *M. sedula* 16S rRNA was selected as a reference gene, since its expression was found to be the most stable under the experimental conditions used. Primers used are listed in Table S1.

### 2.3. PolyP Extraction and Quantification

PolyP was extracted from 1 mL *M. sedula* cultures, essentially as described by Ault-Riché et al. [23]. To quantify PolyP, 30 *μ*L of the extracted samples were mixed with 30 *μ*L of 2 N HCl, and polyP was hydrolyzed by incubation for 30 min at 95°C. Finally, the solution was neutralized with 1.5 M Tris and inorganic phosphate was quantified using the EnzChek Phosphate Assay kit (Invitrogen).

### 2.4. Cloning of PPX*_Msed_* and Generation of Site-Directed Mutants

The gene *Msed_0981* was amplified from the genomic DNA from *M. sedula* DSM 5348^T^ and subcloned in the pGEM-T Easy vector (Promega). After digestion with *NdeI* and *XhoI*, the fragment obtained was cloned in the pET28a-TEV expression vector, which is a derivative of pET28a (Invitrogen) containing the recognition and cut sites for the TEV protease from Tobacco Mosaic Virus. E111A, E112A, and E113A PPX*_Msed_* mutants were carried out by site-directed ligase-independent mutagenesis (SLIM) as described previously [24]. All primers used are listed in Table S1. To purify the wild-type and modified proteins, *E. coli* BL21 (DE3) was transformed with the corresponding plasmids.

### 2.5. Purification of Recombinant PPX*_Msed_* and PPX Activity Assay

*E. coli* cells carrying the plasmid with the recombinant *ppx* gene were grown at 37°C in 200 mL of LB medium until the OD_600_ reached 0.6. Cells were then incubated for 4 h at 37°C, in the presence of 1 mM IPTG, and harvested by centrifugation for 15 min at 1500 ×g. The pellet was resuspended in 30 mL of binding buffer (40 mM imidazole, 0.5 M NaCl, 20 mM Tris-acetate, pH 7.0, 20 mM MgCl_2_, and 175 mM KCl), and cells were lysed by sonication in an ice bath (20 cycles of 20 s pulses, each with 40 s intervals between each cycle). The soluble fraction was separated by centrifugation for 20 min at 16,000 ×g, filtrated through PVDF filters (0.22 *μ*m pore) and loaded onto a pre-equilibrated column containing 1 mL of Profinity IMAC Ni-charged resin (Biorad). After washing the column with binding buffer, proteins were eluted with 1 mL of elution buffer (600 mM imidazole, 0.5 M NaCl, 20 mM Tris-acetate, pH 7.0, 20 mM MgCl_2_, and 175 mM KCl). Fractions containing the purified PPX were pooled and dialyzed against the reaction buffer (50 mM Tris-acetate, pH 7.0, 175 mM KCl, and 1 mM MnCl_2_). To quantify PPX activity, commercial PolyP of different lengths was used. PolyP_45_ and PolyP_75_ were purchased from Sigma (Darmstadt, Germany), and PolyP_700_ was purchased from Kerafast (Boston, MA). PPX activity assay mix contained 50 nmol of PolyP (expressed as total P_i_) and 200 ng of purified recombinant PPX*_Msed_* in the reaction buffer. The reactions were incubated for 30 min at 65°C. PPX activity was determined by analyzing the release of P_i_ from PolyP. P_i_ liberated was measured by using the EnzCheck phosphate assay kit (Invitrogen), and the amount of phosphate present in a reaction done in the absence of the enzyme was subtracted as a blank. Enzymatic polyP degradation was visualized by UREA-PAGE as described before [25]. Electrophoresis was run at 300 V for 1.5 h. The remaining PolyP was stained with a solution containing 0.05% toluidine blue, 25% methanol, and 5% glycerol for 20 min. Finally, the gel was washed with 25% methanol and 5% glycerol to eliminate the excess of toluidine blue.

### 2.6. Homology Protein Modeling of PPX*_Msed_*

A model for the PPX*_Msed_* structure was built including residues 1 to 298, by using homology modeling as implemented in Modeller v9.16 [26]. *Aquifex aeolicus* PPX structure 1T6C was used as the template, and Clustal X was used to align the protein sequences [27]. The coordinates for residues G143, S146, and E148 in the 1T6C structure (G136, S139, and E141 in PPX*_Msed_*) were fixed during the modeling, to maintain their configuration in the final PPX*_Msed_* model.

### 2.7. Transmission Electron Microscopy and Energy-Dispersive X-Ray Microanalysis (EDX)

To visualize *M. sedula* DSM 5348^T^ cells and their electron-dense granules, samples were prepared for electron transmission microscopy as described before [28]. *M. sedula* cells were harvested by centrifugation for 15 min at 1500 ×g and washed by centrifugation for 1 min at 10,000 ×g, with the M88 medium. The pellet was resuspended in M88 medium to a concentration of 1 × 10^9^ cells/mL, and 10 *μ*L of this suspension was placed over nickel grids. The excess of liquid was absorbed with filter paper and grids were vacuum-dried. Unstained cells were observed with a Titan 80–300 kV S/TEM transmission electronic microscope. To determine the elemental composition of the samples, the grids were analyzed by energy-dispersive X-ray microanalysis (EDX), as described before [10, 11].

### 2.8. Statistical Analysis

Statistically significant differences of transcriptional data, comparing means regardless of time and treatments, were determined using two-way ANOVA and the post hoc Tukey HSD test (GraphPad Software Inc.). Enzymatic data were analyzed using a one-way ANOVA, followed by Tukey's test with the same program. *P* values of <0.05 were considered as significant.

## 3. Results and Discussion

### 3.1. Response of Adapted and Nonadapted *M. sedula* DSM 5348^T^ to a Copper Challenge

PolyP has been associated with copper resistance in *A. ferrooxidans* [10] and *S. metallicus* [11], since their high PolyP levels decreased when cells were shifted to a medium containing CuSO_4_. Although it has been previously shown that *M. sedula* DSM 5348^T^ accumulates electron-dense granules [13], the chemical composition of these structures has not been confirmed. Therefore, an elemental analysis of these granules was carried out by electron microscopy coupled with EDX, and the percentages of phosphorous were estimated in different areas of the cell (Figure S1). The 9.43% of phosphorous present in the granule area, compared with 0.07% in the cytoplasm, strongly suggests that these electron-dense granules contain PolyP. Consequently, *M. sedula* DSM 5348^T^ could also have a PolyP-based copper-resistance mechanism as that one proposed for *S. metallicus* and *A. ferrooxidans* [10, 11].

**Figure 1:**
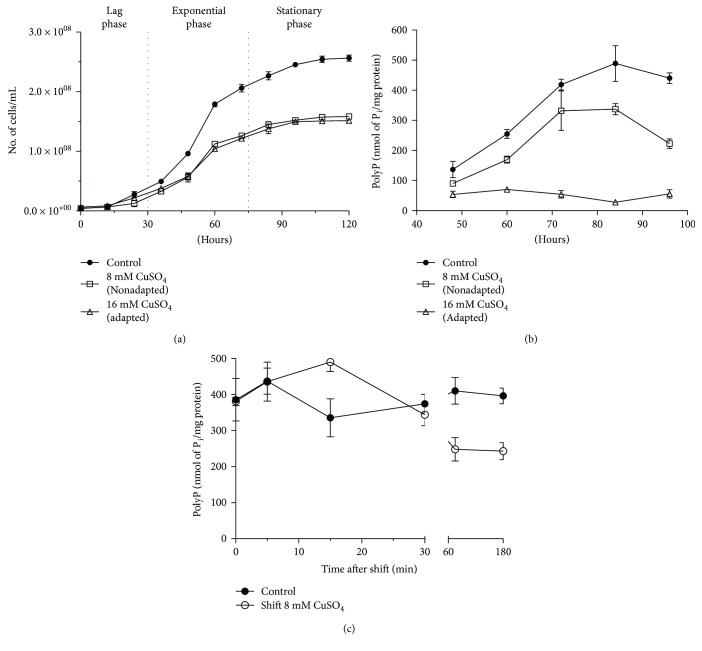
Effect of copper ions in the growth and PolyP levels of *M. sedula* DSM 5348^T^. Growth curves (a) and polyP levels (b) were determined at the indicated copper concentrations added upon inoculation. Cells previously grown up to the late exponential phase were shifted to grow at 8 mM CuSO_4_ at time 0, and polyP levels were immediately determined thereafter at the indicated times after the shift (c).

To evaluate whether PolyP levels changed when *M. sedula* DSM 5348^T^ cells were exposed to copper ions, copper-adapted and nonadapted cells were grown in the presence or absence of the metal.  Figure 1(a) shows that growth of nonadapted *M. sedula* DMS 5348^T^ cells was 50% inhibited when exposed to 8 mM copper, as it has been previously described [29]. In addition, the growth of adapted cells exposed to 16 mM was also half of that observed for cells not exposed to copper.

The levels of PolyP were quantified at different times of growth (Figure 1(b)). *M. sedula* DSM 5348^T^ cells accumulated high amounts of PolyP, reaching up to 450 nmol of PolyP/mg of protein. A 20% decrease in PolyP levels was observed in the nonadapted cells exposed to 8 mM CuSO_4_ compared to control cells. Moreover, PolyP levels of the adapted cells were very low (less than 50 nmol of P_i_/mg of protein), and these levels remained unchanged during the entire growth curve (Figure 1(b)). Therefore, synthesis and hydrolysis of PolyP could be continuously occurring in copper-adapted cells, and as a consequence, the polymer molecules would not accumulate under those conditions. Previously, it was reported that when *Sulfolobus metallicus* (another member of the Sulfolobales) cells are shifted to copper sulfate concentrations up to 100 mM, there was a rapid decrease in their exopolyphosphatase (PPX) activity. This was concomitant in time with a decrease in their polyP levels and a stimulation of a phosphate efflux. In addition, copper in the range of 10 micromolars greatly stimulated PPX activity in cell-free extracts from *S. metallicus* [11]. These results supported a possible role of polyphosphate metabolism in copper resistance, in the members of the genus *Sulfolobus*. It is expected that *M. sedula* has a similar behaviour, although this does not discard a possible copper damage in the unknown archaeal PPK enzyme.

The basal PolyP level remaining in the cells, in the adapted condition, may be required to respond as an inorganic protein chaperone, a role recently described for PolyP in cells subjected to oxidative stress [30]. On the contrary, PolyP levels decreased sharply to around 40% by 60 min after an 8 mM CuSO_4_ shock (Figure 1(c)), similar to what was described before for *S. metallicus* [11] and *A. ferrooxidans* [10]. The results in Figure 1 suggest that PolyP and PPX may also have a role in copper homeostasis in *M. sedula* DSM 5348^T^.

### 3.2. *Msed_0891* Encodes for a Functional PPX Capable of Hydrolyzing PolyP

**Figure 2:**
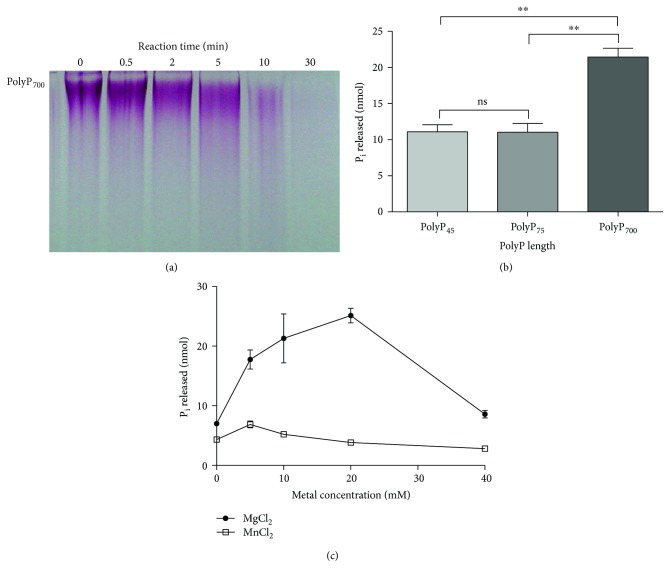
The gene *Msed_0891* encodes for a functional exopolyphosphatase. (a) *In vitro* enzymatic PPX activity assay of the protein encoded by *Msed_0891.* PPX*_Msed_* was incubated with PolyP_700_ as described in Materials and Methods at 65°C for the times indicated. The remaining PolyP in the samples was visualized by UREA-PAGE. (b) Substrate preference of PPX*_Msed_.* The enzyme was incubated with the PolyP of different chain lengths indicated, as described in Materials and Methods. (c) Effect of MgCl_2_ and MnCl_2_ in PPX activity. PPX*_Msed_* activity was determined at the indicated metal concentrations (^∗∗^ *P* < 0.01).

It was suggested that PolyP hydrolysis could be carried out by a putative PPX*_Msed_* encoded by *M. sedula* DSM 5348^T^ gene *Msed_0891* [8]. The decrease in PolyP levels after a copper shock is most likely due to its hydrolysis by this putative enzyme. To evaluate whether *Msed_0891* encoded for a functional PPX, the gene was expressed in *E. coli* and the corresponding protein was purified. PPX*_Msed_* was a functional PPX, able to hydrolyze PolyP *in vitro*, as seen by its disappearance from the reaction by using gel electrophoresis (Figure 2(a)). The optimal temperature for the enzyme was 65°C (data not shown), and it had a preference for long-chain PolyP of 700 residues of P_i_ (Figure 2(b)). It has been reported that the PPXs from *E. coli* (PPX*_Eco_*) and *P. aeruginosa* require Mg^2+^ as a cofactor for their activity [14, 31], whereas the *S. solfataricus* enzyme requires Mn^2+^ [15]. The activity of PPX*_Msed_* increased 3.6-fold in the presence of 20 mM MgCl_2_ (Figure 2(c)), and no effect on its activity was observed when using MnCl_2_ (Figure 2(c)).

**Figure 3:**
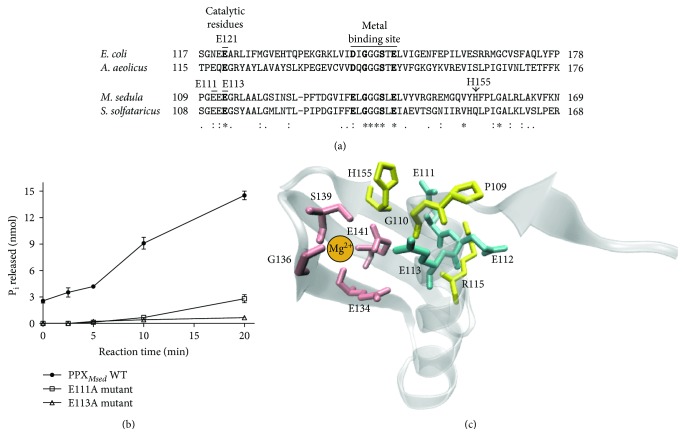
Catalytic residues of the possible active site of PPX*_Msed_*. (a) Protein sequence alignment of the residues in the potential active site of *M. sedula*, *S. solfataricus*, *A. aeolicus*, and *E. coli* PPXs. The residues that have been proven essential for catalysis in PPX*_Eco_* are highlighted in bold [32, 33] (asterisk indicates fully conserved residue, colon indicates strongly similar properties, and period indicates weakly similar properties). (b) Activity of PPX*_Msed_* and the E111A and E113A mutants. (c) Cartoon representation of the possible active site of PPX*_Msed_*. Amino acids in the metal binding site are shown in pink, the mutated residues are shown in cyan, and residues near E111 are shown in yellow.

The active site of PPX*_Eco_* is formed by a catalytic E121 and four residues that coordinate the Mg^2+^ metal cofactor in a metal-binding site: D141, G143, S146, and E148 (Figure 3(a)) [32, 33]. An alignment between the PPXs from *E. coli*, *S. solfataricus*, and *M. sedula* revealed that all the amino acid residues of their putative active sites were conserved, except for D141 that was substituted by a glutamic acid in some of the crenarchaeal proteins (Figure 3(a) and Figure S2). These residues corresponded to E113, E134, G136, S139, and E141 in PPX*_Msed_*. Additionally, E112 was also conserved in the three species, and E111 was only conserved in the PPXs of crenarchaeota. To evaluate the possible role of the residues in the active site, E111, E112, and E113 were replaced by alanine by site-directed mutagenesis, and the activity of the mutants was evaluated. Mutants E111A and E113A had no PPX activity, suggesting that these residues are essential for catalysis in PPX*_Msed_* (Figure 3(b)). On the contrary, the PPX activity of mutant E112A was the same compared to that of the wild-type enzyme (not shown). These results suggest that both E111 and E113 could be catalytic residues in PPX*_Msed_*. Theorizing about a structural connection between E111, E113, and a possible catalytic mechanism, a homology model of PPX*_Msed_* was generated and compared to the crystallized PPX*_Eco_* (PDB: 1U6Z). The *Aquifex aeolicus* PPX (PPX*_Aaeo_*) (PDB: 1T6C) was used as the template because it has 28% identity with PPX*_Msed_*, and the structure has a Ca^2+^ ion in the metal-binding site of the active site, resembling the divalent cation expected for PPX*_Msed_*. Considering the difference in the number of amino acid residues between the two proteins (315 in PPX*_Aaeo_* versus 420 in PPX*_Msed_*) and the fact that the sequences and structures of the active sites are conserved in most of the crystallized PPXs so far, solely the first 298 residues of PPX*_Msed_* were modeled, and only the active site was analyzed (Figure 3(c)). The model anticipated that E113 was the closest residue to the metal-binding site in PPX*_Msed_* and it could be the catalytic residue. E111 was predicted to be located 2.66 Å from H155 in PPX*_Msed_*. E111 could indirectly stabilize the metal-binding site, by preventing the interaction between H155 and E141 that could disturb the proper coordination of the metal. On the other hand, the side chain of E112 points in the opposite direction of the metal-binding site. This could structurally explain why the mutation of this residue did not alter the activity of PPX*_Msed_*. Further biophysical analyses should be carried out to prove these hypotheses.

**Figure 4:**
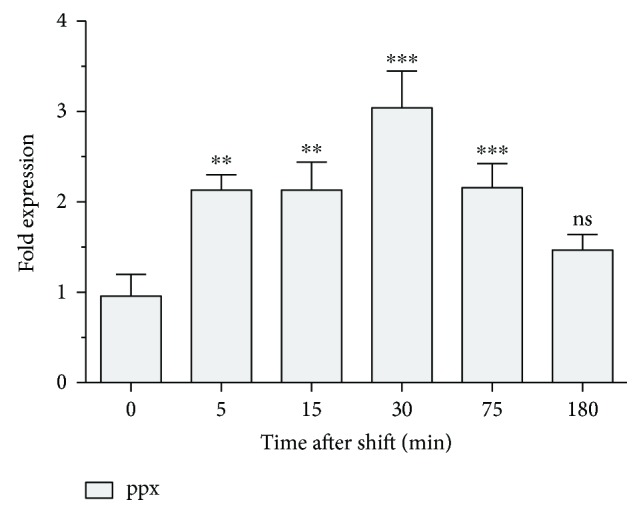
Transcriptional levels of *ppx* gene increase significantly in *M. sedula* DSM 5348^T^ cells after a copper shift. Relative transcriptional levels of *ppx* in *M. sedula* cells exposed to an 8 mM copper shift were measured by qRT-PCR. (^∗∗^ *P* < 0.01; ^∗∗∗^ *P* < 0.001; ns: nonsignificant).

In terms of transcriptional regulation, it would be possible that *ppx* levels increase after a copper shock, since PolyP amounts were sharply reduced under that stressing condition (Figure 1(c)). It was previously reported that transcriptional levels of *ppx* did not change significantly, after 1 h shock of *M. sedula* DSM 5348 T, in the presence of metals such as copper, zinc, nickel, uranium, and cobalt as determined by microarray analyses [29]. However, it was not assessed whether *ppx* was induced at earlier times after the shock. Accordingly, transcriptional levels of *ppx* were quantified by qRT-PCR at different times after an 8 mM CuSO_4_ shock (Figure 4). It was found that *ppx* was upregulated during the first 75 min after the metal shift, with a peak in its expression 30 min after the shift. The results presented here suggest that PPX*_Msed_* could play an important role in short-term copper resistance after a metal shock, possibly being one of the first copper determinants used to respond to copper stress.

It is known that PPX*_Eco_* is inhibited *in vitro* by 20 mM P_i_ [14]. PPX*_Msed_* was also inhibited in vitro in the presence of 1 mM P_i_ (data not shown). This inhibition may be very important in vivo, since PolyP hydrolysis stimulated by the presence of the metal could come to an end.

### 3.3. Relative Transcriptional Expression of *copTMA* Genes in Adapted and Nonadapted *M. sedula* DSM 5348^T^ Challenged to Copper

**Figure 5:**
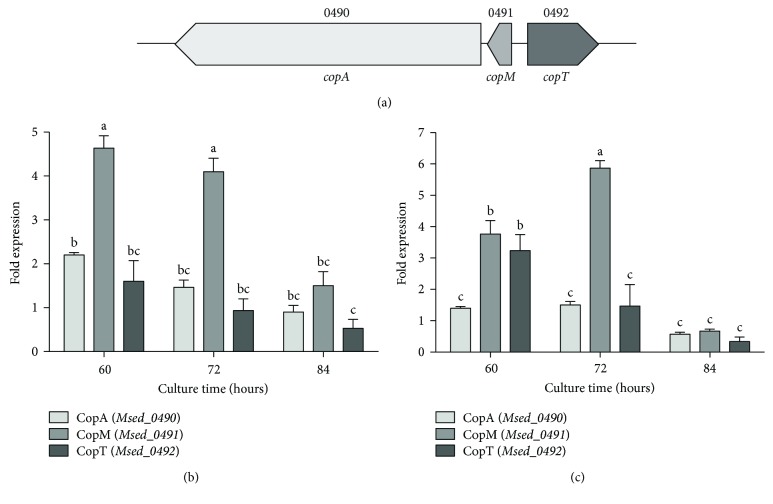
Transcriptional levels of *copA*, *copM*, and *copT* genes at different points in the growth phase of *M. sedula* DSM 5348^T^ cells grown in the presence of copper ions. (a) Diagram of the *copTMA* in *M. sedula* DSM 5348^T^. Relative gene expression was quantified by qRT-PCR. (b) Nonadapted cells exposed to 8 mM CuSO_4_. (c) Adapted cells exposed to 16 mM CuSO_4_. Error bars indicate standard deviations based on three different experimental values. Values with different letters differ significantly (*P* < 0.05).

Other proteins that are involved in copper resistance in *M. sedula* DSM 5348^T^ are CopA (*Msed_0490*), CopM (*Msed_0491*), and CopT (*Msed_0492*) [8]. CopA is a P-type ATPase efflux pump, CopM is a putative metallochaperone, and CopT is a transcriptional regulator homolog to CopR. All these genes are functional in this archaeon [9]. In *S. solfataricus*, these genes are cotranscribed constitutively, as an operon. However, only the transcription of *copM* and *copA* was also upregulated in the presence of copper, due to the activation of a second promoter upstream of the constitutive *copTMA* promoter [34, 35]. While in *S. solfataricus*, the three genes form a cluster in the same direction; *copT* in *M. sedula* DSM 5348^T^ is orientated opposite to the other genes (Figure 5(a)) [9]. In terms of regulation, microarray analyses revealed that the expression levels of *copA* and *copM* increased and *copT* levels decreased after 60 min of a 4 or 8 mM CuSO_4_ shift in *M. sedula* DSM 5348^T^ [29]. This kinetic behaviour is equivalent to that observed for *ppx* (Figure 4).

Short-term copper effects can be analyzed with a shock condition, since the microorganism is exposed to the metal for briefer periods compared with its generation time. On the contrary, long-term effects can be analyzed during the growth of nonadapted cells for intervals longer than a generation time, in adapted cells, in which the microorganisms have been exposed to the metal for several generations. To assess for a possible differential timing in the expression of these genes, during long-term copper exposure in *M. sedula* DSM 5348^T^, relative transcriptional levels of genes *Msed_0490*, *Msed_0491*, and *Msed_0492* were quantified by qRT-PCR in copper-adapted and nonadapted cells grown at exponential (60 h), late exponential (72 h), and stationary (84 h) phases (Figure 5). In general, gene expression of these determinants was higher at the exponential phase (Figures 5(b) and 5(c)). In the nonadapted condition, when cells were exposed for the first time to 8 mM CuSO_4_, the expression of *copM* increased 4.5-fold at 60 and 72 h in exponential phase (Figure 5(b)). In cells adapted to copper exposure, the transcriptional levels of *copM* were up to three times higher than that of the other genes in exponential phase and had a peak in its expression at 72 h (Figure 5(c)). These results suggest that the genes analyzed are regulated differently in response to copper ions and that their expression might also be controlled by distinct internal copper-responsive promoters and transcription regulators, as it occurs in *S. solfataricus* [35].

### 3.4. Possible *Pho84*-Like Phosphate Transporters Are Upregulated Differently in *M. sedula DSM 5348^T^* Exposed to Copper

The model for copper resistance in microorganisms mediated by PolyP proposes that the P_i_ released from PolyP by PPX could complex metal ions. These complexes would be transported out of the cell by H^+^-phosphate symporters [13, 16, 17], such as the low-affinity phosphate transporter PitA from *E. coli* [18]. Recently, a *pitA*-homolog gene was found in *M. sedula* CuR1, which is truncated in the “wild-type” strain DSM 5348^T^ [19]. As already mentioned, there are no *pitA*-homologs in some acidophilic species, such as the bacterium *A. ferrooxidans* and the archaeons *S. metallicus* and *F. acidarmanus* [4]. In those microorganisms, it has been proposed that proteins orthologous to the *Pho84* phosphate transporter from *Saccharomyces cerevisiae* could carry out the role of PitA, transporting metal-phosphate complexes outside the cell [10, 11]. The copper MIC value (8–16 mM) of *M. sedula* DSM 5348^T^ is much lower than that of CuR1 mutant (160 mM Cu) [19]. Therefore, the *pho84*-like phosphate transporters present in the strain DSM 5348^T^ would not entirely replace its lack of PitA, in eliminating Cu-phosphate complexes. As suggested before, some unidentified transport protein could be responsible for excluding these complexes instead of PitA [19].

It is also important to consider that an excess of Pi entering the cells through the PitA system or other phosphate transporters would be toxic to the microorganisms. The *pho84*-like phosphate transporters present in *M. sedula* DSM 5348^T^ may also have a role in eliminating the extra Pi generated from polyP.

**Figure 6:**
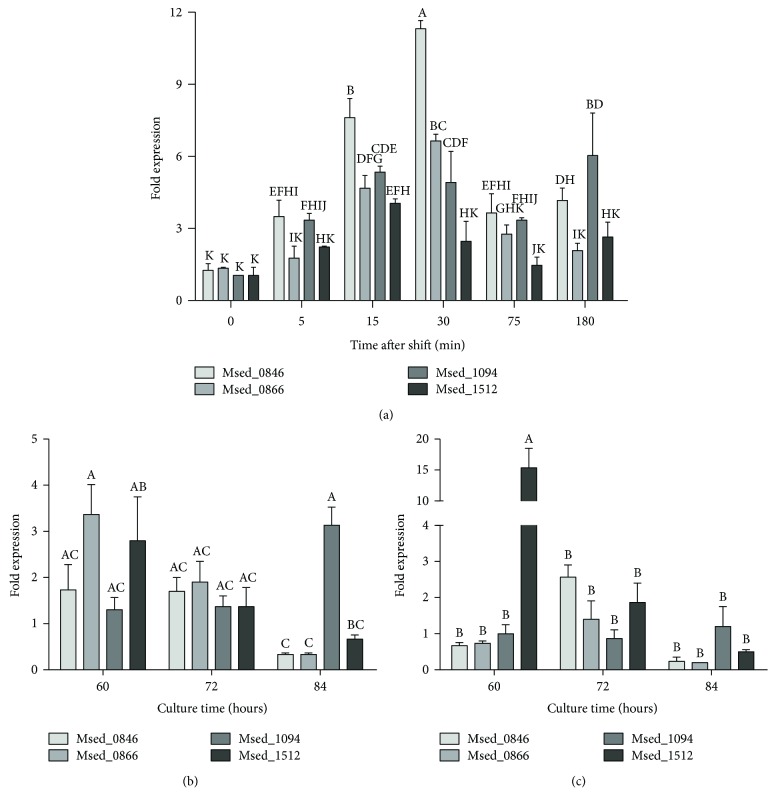
Differential transcriptional expression of putative *pho84*-like genes in *M. sedula* DSM 5348^T^ cells subjected to copper. (a) Nonadapted cells exposed to an 8 mM copper shift during the indicated times. (b) Nonadapted cells exposed to 8 mM CuSO_4_ during the indicated culture times. (c) Adapted cells exposed to 16 mM CuSO_4_. Values with different letters differ significantly (*P* < 0.05).

*Saccharomyces cerevisiae Pho84* is a high-affinity H^+^-phosphate symporter functional only at acidic pH [36]. In the genome of *M. sedula* DSM 5348^T^, four genes have been annotated as putative *Pho84*-like phosphate transporters: *Msed_1512*, *Msed_1094*, *Msed_0866*, and *Msed_0846* [8]. To evaluate whether the transcription of these genes is copper-responsive, their transcriptional levels were quantified by qRT-PCR in *M. sedula* DSM 5348^T^ cells shifted to an 8 mM CuSO_4_ shock (Figure 6(a)). The four putative *Pho84*-like genes were upregulated after the metal shift, with a maximum expression at 30 min after the shock. *Msed_0846* showed the highest increase after the shift, suggesting it could be the most important transporter in an immediate copper response in *M. sedula* DSM 5348^T^. This behaviour is similar to that of the *ppx* gene already seen in Figure 4, suggesting a possible common regulation mechanism for this group of genes.

Moreover, it has not been previously reported whether this copper-resistance PolyP-based mechanism has a role in long-term copper resistance in *M. sedula*. To evaluate the possible role of *pho84*-like genes in long-term copper response in *M. sedula* DSM 5348^T^, the transcriptional expression of these genes was assessed by qRT-PCR during exponential (60 h), late exponential (72 h), and stationary (84 h) phases of nonadapted and adapted cells. Nonadapted cells exposed to 8 mM CuSO_4_ showed differences in the expression of the putative phosphate transporters during growth (Figure 6(b)). Transcriptional levels of *Msed_0866* and *Msed_1512* were maximal at 60 h and 72 h of exposure to the metal (in exponential phase), whereas *Msed_1094* had its peak in stationary phase at 84 h of copper exposure. The expression of *Msed_0846* did not change significantly in both nonadapted and adapted cells. In *M. sedula* DSM 5348^T^ cells adapted to 16 mM CuSO_4_, the transcriptional levels of the putative transporter encoded by *Msed_1512* increased 15-fold at exponential phase (Figure 6(c)). The transcriptional levels of the other possible transporters were not significantly different in the adapted cells. These results suggest that the 4 genes are upregulated by the presence of copper, but their “transcriptional timing” could be different in the nonadapted growth condition. Possibly, *Msed_0866* and *Msed_1512* could have predominant roles during exponential phase and *Msed_1094* could be the most important putative transporter in stationary phase. *Msed_0846* might have a role in the immediate defense mechanism against a copper stress, as in the case of a metal shock. On the contrary, under a constant high copper exposure, such as 16 mM CuSO_4_ in the adapted condition, *Msed_1512* could be by far the most important copper-responsive transporter during the exponential phase of growth.

## 4. Conclusions

**Figure 7:**
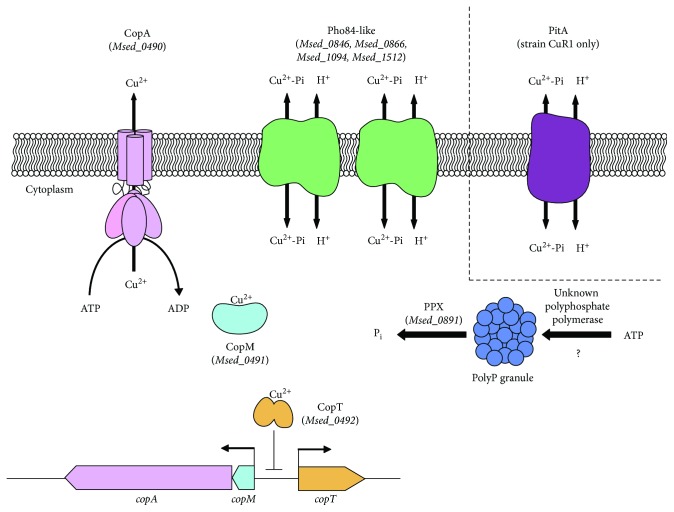
Cartoon working model for copper resistance in *M. sedula*. In *M. sedula* DSM 5348^T^, copper ions could bind to the transcriptional repressor CopT (*Msed_0492*) and prevent the binding of this protein to the DNA to activate the transcription of *copA* (*Msed_0490*), *copT*, and *copM* (*Msed_0491*) genes. CopM is a possible Cu-binding protein that could sequester copper ions in the cytoplasm. CopA is a putative P-type metal-efflux ATPase that could couple the efflux of copper ions from the cytoplasm and the energy from ATP hydrolysis. On the other hand, PPX (*Msed_0891*) could hydrolyze PolyP to generate P_i_ that could complex copper ions. These complexes could be transported outside the cell, together with H^+^ by the H^+^-phosphate symporters, such as the *Pho84*-like phosphate transporters (*Msed_0846*, *Msed_0866*, *Msed_1094*, and *Msed_1512*) in both strains DSM 5348^T^ and CuR1. This last strain has an additional PitA phosphate transporter that could enhance the efflux of complexes and would explain its higher copper resistance. This model is based on the results of the present work and previously reported proposals [8, 9, 11, 13, 19, 29, 34, 35].

The experimental evidence obtained indicates that *M. sedula* strain DSM 5348^T^ can live in 16 mM CuSO_4_ and could have a polyP-based copper-resistance mechanism. The higher transcriptional expression of the four putative *pho84*-like phosphate transporter genes (*Msed_0846*, *Msed_0866*, *Msed_1094*, and *Msed_1512*) in *M. sedula* DSM 5348^T^ grown in the presence of copper suggests that the proteins they code for could carry out the same function of PitA present in other species.  Figure 7 shows a working cartoon model with the possible copper-resistance determinants known in *M. sedula*. The experiments presented here suggest that this model would be a dynamic one and that not all the indicated proteins are needed in the same levels during the different stages of copper resistance. After an immediate exposure to copper ions, as it occurs in the case of a sudden metal shift, all possible determinants studied were overexpressed after the shift. In the nonadapted phase, when the archaea have grown less than 90 h under a copper stress, CopM could have an important role possibly in sequestering metal ions within the cell. *Pho84*-like phosphate transporters have differential expression: while *Msed_0866* and *Msed_1512* seem to be needed preferentially during exponential phase, *Msed_1094* could have a major contribution during stationary phase. In the very late stages of copper resistance, when the cell is adapted to higher concentrations of the metal, *Msed_1512* could be the most important *Pho84*-like phosphate transporter and CopA and CopM could be involved in copper efflux and sequestration of the metal, respectively. The results presented suggest there might be a tight regulation network behind the expression of these genes. Nevertheless, further work will be required to understand better the suggested role for the components involved in copper resistance in *M. sedula*.

## Supplementary Materials

Supplementary 1 Table S1: oligonucleotides used in this work.

Supplementary 2 Figure S1: electron-dense granules of *M. sedula* DSM 5348 T are phosphorous-rich and most likely contain PolyP. A. *M. sedula* DSM 5348 T was analyzed by transmission electron microscopy (TEM). Unstained and unfixed cells taken from M88 medium were placed over nickel grids and examined directly for the presence of electron-dense granules. B. By using TEM coupled to energy dispersive X-ray analysis (EDX) the elemental composition of a granule (1), a cytoplasmic region (2) and an extracellular area (3) were analyzed and the percentage of phosphorous obtained in each case is indicated.

Supplementary 3 Figure S2: protein sequence alignment of the residues present in the possible active sites of archaeal PPXs and PPXEco. Only part of the protein sequence alignment is shown. Residues that could be essential for catalysis in PPXs are highlighted in red. Residue H155 of PPXMsed and its possible homolog residues in the other PPXs are highlighted in blue, and their position is shown with an arrow.
