# Supplementary material for: Inorganic Polyphosphate, Exopolyphosphatase, and Pho84-Like Transporters May Be Involved in Copper Resistance in Metallosphaera sedula DSM 5348T
Source: Archaea. 2018 Mar 5;2018:5251061. doi: 10.1155/2018/5251061 (PMC5859850; doi:10.1155/2018/5251061)
Supplement: Supplementary 1 — Table S1: oligonucleotides used in this work. [file 5251061.f1.pdf]

Supplementary Tables and Figures

Table S1. Oligonucleotides used in this work

Primer	Sequence (5'-3')	Use
PPX fw	CATATGGAGGAAGTTGGAG	Cloning of *Msed_0981*
PPX rv	CTCGAGTCAATAAACACCCAAGGG	Cloning of *Msed_0981*
mutPPX fw ex	AGACTCGCAGCCCTAGGATCCATA	Mutagenesis
mutPPX rv ex	TATGATCCTAATATCGTGACCCAG	Mutagenesis
mutPPXE112A fw	CCAGGAGAAGCGGAGGGAAGACTCGCAGCCCTAGGATCCATA	Mutagenesis E112A
mutPPXE112A rv	TCCCTCCGCTTCTCCTGGTATGATCCTAATATCGTGACCCAG	Mutagenesis E112A
mutPPXE111A fw	CCAGGAGCGGAGGAGGGAAGACTCGCAGCCCTAGGATCCATA	Mutagenesis E111A
mutPPXE111A rv	TCCCTCCTCCGCTCCTGGTATGATCCTAATATCGTGACCCAG	Mutagenesis E111A
mutPPXE113A fw	CCAGGAGAAGAGGCGGGAAGACTCGCAGCCCTAGGATCCATA	Mutagenesis E113A
mutPPXE113A rv	TCCCGCCTCTTCTCCTGGTATGATCCTAATATCGTGACCCAG	Mutagenesis E113A
Msed_1512 fw	TTACCTTGCCTCCAGATCTTGCCT	qRT-PCR Msed_1512
Msed_1512 rv	AGGCGTTTCTGGGACCTTTCTTCT	qRT-PCR Msed_1512
Msed_1094 fw	TGGAGGTATTCCTTCGCGTTGGA	qRT-PCR Msed_1094
Msed_1094 rv	TTGGGTTGGGATTCCATCTCCCTT	qRT-PCR Msed_1094
Msed_0866 fw	ACTGCAGGTTGTAGGATTCGTGGT	qRT-PCR Msed_0866
Msed_0866 rv	TGCAGTCCGTAAAGGAAGAAGGCT	qRT-PCR Msed_0866
Msed_0846 fw	AGTGCAGGCCTAAACGACAGACTT	qRT-PCR Msed_0846
Msed_0846 rv	TGCCGAAGAGGAAAGCAAAGAGGA	qRT-PCR Msed_0846
Msed_0981 fw	ATCTTTGAGCTGGGAGGAGGTTCA	qRT-PCR Msed_0981
Msed_0981 rv	GGCAACCAGCTTGGCAATGAGTAA	qRT-PCR Msed_0981
Msed_0490 fw	CGCAAGTGAAACACCACCCATGAA	qRT-PCR Msed_0490
Msed_0490 rv	ACCTTTGGGCTCCCGTGTTATCTT	qRT-PCR Msed_0490
Msed_0491 fw	AGTTAAGGAGACCTCGGCCTACAA	qRT-PCR Msed_0491
Msed_0491 rv	AAAGGCCGTCTTACAGTGTGGACT	qRT-PCR Msed_0491
Msed_0492 fw	CAGTGGCAAAGATCCTTCGT	qRT-PCR Msed_0492
Msed_0492 rv	CCGCTATCTCTCCTCCACAG	qRT-PCR Msed_0492
16S fw	GCCGCAAGGCTGAAACTTAAAGGA	qRT-PCR 16S
16S rv	AACCGGACATTTCACAACACGAGC	qRT-PCR 16S
